# Combined effect of daily physical activity and social relationships on sleep disorder among older adults: cross-sectional and longitudinal study based on data from the Kasama study

**DOI:** 10.1186/s12877-021-02589-w

**Published:** 2021-11-03

**Authors:** Jaehoon Seol, Jaehee Lee, Koki Nagata, Yuya Fujii, Kaya Joho, Korin Tateoka, Taiki Inoue, Jue Liu, Tomohiro Okura

**Affiliations:** 1 Faculty of Health and Sport Sciences, University of Tsukuba, 1-1-1 Tennodai, Tsukuba, Ibaraki 305-8574 Japan; 2 International Institute for Integrative Sleep Medicine (WPI-IIIS), University of Tsukuba, 1-1-1 Tennodai, Tsukuba, Ibaraki 305-8575 Japan; 3 Japan Society for the Promotion of Sciences, 5-3-1 Kojimachi, Chiyoda, Tokyo, 102-0083 Japan; 4 Master’s Program in Physical Education, Health and Sports Sciences, Graduate School of Comprehensive Human Sciences, University of Tsukuba, 1-1-1 Tennodai, Tsukuba, Ibaraki 305-8574 Japan; 5 Doctoral Program in Public Health, Graduate School of Comprehensive Human Sciences, University of Tsukuba, 1-1-1 Tennodai, Tsukuba, Ibaraki 305-8574 Japan; 6 Physical Fitness Research Institute, Meiji Yasuda Life Foundation of Health and Welfare, 150 Tobukimachi, Hachioji, Tokyo, 192-0001 Japan; 7 Doctoral Program in Human Care Science, Graduate School of Comprehensive Human Sciences, University of Tsukuba, 1-1-1 Tennodai, Tsukuba, Ibaraki 305-8574 Japan; 8 J-Stretch Association, 1132-1-504 Ochikawa, Hino-city, Tokyo 191-0034 Japan; 9 Doctoral Program in Physical Education, Health and Sports Sciences, Graduate School of Comprehensive Human Sciences, University of Tsukuba, 1-1-1 Tennodai, Tsukuba, Ibaraki 305-8574 Japan; 10 R&D Center for Tailor-Made QOL, University of Tsukuba, 1-2 Kasuga, Tsukuba, Ibaraki 305-8550 Japan

**Keywords:** Insomnia, Inactive, Social isolation, Exercise, Depression

## Abstract

**Background:**

This study investigated whether daily physical activity of older adults, combined with social relationships, is associated with the risk of sleep disorder. Further, it determined whether a high level of one variable with a low level of the other, leads to a significantly lower risk of sleep disorder than low levels of both.

**Methods:**

The sample comprised 1339 community-dwelling older Japanese adults: 988 in Study 1 and 351 in Study 2. The level of daily physical activity and range of social relationships were assessed using the Physical Activity Scale for the Elderly and the Lubben Social Network Scale, respectively. The Pittsburgh Sleep Quality Index was used to assess sleep disorder. To test the combined relationships and effects in Studies 1 and 2, the medians for the respective scores of each of the following four groups that the participants were categorized into, were calculated: (1) low activity group with low social relationships, (2) low activity group with high social relationships, (3) high activity group with low social relationships, and (4) high activity group with high social relationships. After adjusting for potential confounders, a logistic regression analysis was conducted in Study 1. After adjusting for potential confounders, a Cox proportional hazards regression analysis was conducted in Study 2.

**Results:**

Study 1 revealed that the high activity group with high social relationships showed a significantly lower risk of sleep disorder (ORs: 0.585, 95% CI: 0.404–0.847) than the low activity group with low social relationships. Study 2 also revealed that the high activity group with high social relationships showed a significantly lower prevalence of sleep disorder (HRs: 0.564, 95% CI: 0.327–0.974) than the low activity group with low social relationships.

**Conclusions:**

Our findings suggest that for older adults with high social relationships, being physically active is favorably associated with sleep quality. However, a high level of one variable with a low level of the other has not been confirmed in improving sleep quality among older adults.

## Background

Sleep disorder is common in later life and various epidemiological studies have reported that it affects 36–70% of community-dwelling older adults [1, 2]. The prevalence of sleep disorders increases dramatically as people reach older adulthood [3], and sleep disorders are strongly linked to risks of mortality, falls, cognitive impairment, and depression [4–7].

The American Geriatrics Society recommends the use of nonpharmacological tools to improve sleep quality [8]. Exercise is a representative nonpharmacological intervention, and high levels of daily physical activity have been found to be associated with a lower prevalence of sleep disorder in older adults; however, the effects of exercise in this regard depend on the intensity, duration, and time of day at which it is performed [9–11]. In addition, some epidemiological studies have suggested that high levels of housework and/or work-related activities are associated with a lower prevalence of sleep disorder [12, 13]. These studies suggest that, similar to exercise, housework and/or work-related activities are positively associated with thermoregulation and/or regulation of the circadian system, which consequently improves sleep quality in older adults [12, 13]. Physical activity, including exercise, is a free or low-cost method of promoting health benefits, especially physical and cognitive function, mood, and sleep, and has a low risk of side effects in older adults [14]. Sleep disorder is associated with poorer physical functioning among older adults [15, 16]; however, the mechanism by which disturbed sleep impacts physical functioning is currently unknown. It is possible that the relationship between physical activity and sleep quality is mediated by physical functioning [16].

Previous studies have found that a narrow range of social relationships is associated with an increased risk of mortality [17–19]; this is because long-term social isolation can create chronic stress [17]. Conversely, some epidemiological studies have reported that a wide range of positive social relationships (e.g., supportive ties) is related to lower prevalence of sleep disorder [20, 21]. An epidemiological study revealed that older adults who have a large number of positive social relationships for at least 15 years have better sleep quality than those with shorter-duration relationships [21]. Furthermore, combining low-intensity physical activity with social interaction has been found to increase the prevalence of slow-wave sleep (i.e., deep sleep) and improve cognitive function among older adults [22]. In later life, older adults naturally have fewer social relationships (e.g., as a result of retirement, bereavement); the above findings suggest that older adults should seek to counteract this by endeavoring to maintain or expand their range of social relationships, as this is an important factor for their quality of life.

Physical activity and social relationships for older adults are positively related to not only physical and cognitive functions and mental health but also sleep quality. Both variables are positively correlated [23]; however, there is insufficient previous research considering the confounding factors for each of these variables [9, 10, 20]. Furthermore, some older adults lack either social relationships or physical activity, or both. Considering this finding and previous results, we hypothesized that the prevalence of sleep disorder is lower among people with a low level of one of these variables (i.e., social relationships or physical activity) and a high level of the other, compared to those who are inactive and have a narrow range of social relationships. Thus, to identify the relationship between sleep quality and a high level of one of these variables with a low level of the other, we examined the combined effect of physical activity and social relationships on the prevalence of sleep disorder among community-dwelling older adults in Japan through a cross-sectional (Study 1) and longitudinal study (Study 2).

## Materials and methods

### Participants and data collection

Both Studies 1 and 2 were based on data from the Kasama Study, which was a community-based cohort study conducted in Japan between 2011 and 2019 [24]. Participants for each year of the study were randomly selected from the Basic Resident Register using the following eligibility criteria: (1) aged 65 years or older, (2) not a recipient of long-term care insurance, and (3) living in Kasama City, Japan. A total of 1094 community-dwelling older adults participated in Study 1, of whom 106 (9.7%) were excluded because of incomplete data. Ultimately, 988 participants were included in the analysis. In Study 2, we excluded 575 older adults who had not participated more than twice during 2011–2019, a further 199 participants who had a sleep disorder, and 25 who had incomplete data. Finally, 351 participants were included in Study 2’s analysis.

### Daily physical activity

The Japanese version of the Physical Activity Scale for the Elderly (PASE) [25] was used to assess participants’ daily physical activity. The PASE is a 12-item questionnaire that measures the average hours per day spent performing leisure-time, housework, and work-related physical activity, respectively, over the previous 7 days. Leisure-time physical activity includes walking; light-, moderate-, and vigorous-intensity recreational activities; and muscle-strength training. Housework includes light and heavy housework, home repair, lawn work or yard care, outdoor gardening, and caring for other people. Finally, work-related physical activity includes paid and volunteer work. These items are weighted based on the intensity of each activity, and the total PASE score is the sum of the 12 weighted items [25]. This study used the PASE total score to determine participants’ overall physical activity levels [26].

### Social relationships

To assess social relationships, we used the Japanese version of the Lubben Social Network Scale-6 (LSNS) [27]. The LSNS assesses social relationships by measuring the strength of respondents’ family ties and friendship ties, respectively, in terms of each of the following three categories: (1) “How many people do you see or hear from at least once a month?” (2) “How many people do you feel sufficiently at ease with such that you can talk with them about private matters?” (3) “How many people do you feel sufficiently close to such that you could call on them for help?” All questions were answered using a six-point Likert scale, where 0 = “none,” 1 = “one,” 2 = “two,” 3 = “three or four,” 4 = “five through eight,” and 5 = “nine or more”. The LSNS score was determined by summing the scores for all six items (thus, it ranges from 0 to 30) [27]. Due to the quantitative estimates of social relationships, this study used continuous data divided into median or tertiles [28, 29].

### Sleep disorder

We used the Pittsburgh Sleep Quality Index (PSQI) to identify sleep disorder; this tool has been used in both clinical research and epidemiological studies [30]. The PSQI measures seven components: subjective sleep satisfaction, sleep efficiency, sleep onset latency, sleep duration, sleep disturbances, use of sleeping medication, and daytime dysfunction. The score for each component is weighted by 0 to 3, and the PSQI global score is determined by summing all items (thus, it ranges from 0 to 21). A previous study revealed that with a cut-off of 5/6, the PSQI global score has a sensitivity of 89.6% and a specificity of 86.5% for identifying cases of sleep disorder [30]. In this study, this cut-off value was used to define an individual having a sleep disorder.

### Physical functions

To assess physical function between the groups in Study 1, we conducted five physical performance tests: grip strength, one-leg standing duration, time-up and go test, 5-m walk test, and 5 times sit-to-stand test. Grip strength was measured twice on each hand by a grip dynamometer (TKK 5401, Takei Scientific Instruments Co., Ltd., Niigata, Japan) and the average of the best values of each hand was adopted. To evaluate static balance ability, we conducted a one-leg standing with eyes open test. Participants put both hands on their waist and gradually raised their preferred foot in front of them to approximately 20 cm above the floor. They maintained this position as long as was possible (up to 60 s). The records were captured twice, and the highest record was adopted. To assess dynamic balance ability, we used the time-up and go test. Participants rose from a chair, walked 3 m as quickly as possible, turned around, walked back, and sat down. We assessed usual gait speed using the 5-m walk test. Participants walked at their typical speed on an 11-m straight course. To eliminate acceleration phases, we calculated walking time between the 3- and 8-m marks of the course. To assess lower-limb muscle strength, we conducted the 5-times sit-to-stand test. Participants sat on a chair with their arms over their chest. They carried out 5 consecutive sit-to-stand cycles as quickly as possible. The time-up and go test, 5-m walk test, and 5 times sit-to-stand test were conducted twice, and the faster record was adopted [31, 32].

### Potential confounders

To identify potential confounders, we referenced previous studies [33] and included measures of age, sex, and body mass index (BMI); use of hypertension, psychotropic, diabetes, and sleep medication, respectively (respondents answered “yes” or “no” for all); medical history of lower back pain, knee pain, and hip pain, respectively (“yes” or “no” for all); alcohol consumption (“drinker” or “non-drinker”); tobacco-smoking status (“current” or “previous/never”); and presence of depressive syndrome (assessed using the Japanese version of the 15-item Geriatric Depression Scale (GDS) [34]).

### Statistical analysis

#### Cross-sectional study (study 1)

Linear trends in the prevalence of sleep disorder were computed using ordinal scoring for the PASE and LSNS scores, respectively [35]. For both scales, scores were divided into tertiles; for the PASE score, 0–94.3 points (*n* = 329) represented the “first tertile”; 94.5–140.4 points (*n* = 329) represented the “second tertile”; and 140.6–462.3 points (*n* = 330) represented the “third tertile”; meanwhile, for the LSNS score 1–15 points (*n* = 351) represented the “first tertile”; 16–19 points (*n* = 312) represented the “second tertile”; and 20–30 points (*n* = 325) represented the “third tertile.” Additionally, to test the relationship between the combined two factors and sleep disorder, the two factors’ medians were calculated for the respective scores, and these were used to develop the following participant groups: (1) low activity group with low social relationships (*n* = 307); (2) low activity group with high social relationships (*n* = 183); (3) high activity group with low social relationships (*n* = 205); and (4) high activity group with high social relationships (*n* = 293).

To compare the four groups, we used one-way analyses of variance with Bonferroni post-hoc tests for continuous variables and chi-square tests for categorical variables including physical functions. We used logistic regression analysis to examine the association between physical activity and social relationships, individually and in combination with each other, on the risk of sleep disorder. First, in the tests of linear trends, we adopted two models. Model 1 was adjusted for age, sex, BMI, use of hypertension and sleeping medication, smoking and drinking habits, and depressive syndrome. There was a significant correlation between the PASE and LSNS scores (*r* = 0.266, *P* < 0.001). Thus, along with the adjustments contained in Model 1, Model 2 featured additional adjustments for physical activity variables when examining social relationships. Further, additional adjustments for social relationship variables when examining physical activity were applied. Second, only Model 1 was used to examine the combined association between physical activity and social relationships on sleep disorders. We calculated odds ratios (ORs) and 95% confidence intervals (CIs).

#### Longitudinal study (study 2)

Similar to Study 1, to test the combined effect of the two factors, the medians were calculated for the respective scores, and these were used to develop the following participant groups: (1) low activity group with low social relationships (*n* = 106); (2) low activity group with high social relationships (*n* = 66); (3) high activity group with low social relationships (*n* = 69); and (4) high activity group with high social relationships (*n* = 110). To investigate the combined effect of the two factors on sleep disorder, we conducted Cox proportional hazards regression analysis and calculated hazard ratios (HRs) and 95% CIs. The independent variable was the low activity group with low social relationships as the reference, and duration of existence was determined for this cohort. Further, sleep disorder occurrence was initially defined when diagnosed for the first time during the follow-up period. Study 2 adopted only Model 1.

All analyses were performed using IBM SPSS Statistics version 26.0 (IBM Corp., Armonk, NY, USA).

## Results

The mean age of the sample in Study 1 was 73.1 ± 5.5 years, and 56.3% of the participants were female. Individuals with sleep disorder accounted for 36.8% of the total sample. Table 1 shows the demographic characteristics of the four participant groups. The active group with a narrow range of social relationships featured a significantly lower ratio of females, while the active group with a wide range of social relationships featured a significantly higher ratio of females than the other groups. Both high activity and low activity groups with high social relationships (*P* < 0.001) showed significantly lower GDS scores than the low activity with low social relationships. In addition, the high activity group with high social relationships showed a significantly lower GDS score than the high activity group with low social relationships (*P* < 0.001). Naturally, the LSNS and PASE scores significantly differed among the groups because of the participant group formation methods used. Notably, however, the high activity group with high social relationships had a significantly higher PASE score than the high activity group with low social relationships (*P* < 0.001).

**Table 1:** Characteristics of participants

Variables	low activity group with low social relationships ^1^ (*n* = 307)	low activity group with high social relationships ^2^ (*n* = 183)	high activity group with low social relationships ^3^ (*n* = 205)	high activity group with high social relationships ^4^ (*n* = 293)	ANOVA or chi square *P* value	Post Hoc
	MEAN ± SD	MEAN ± SD	MEAN ± SD	MEAN ± SD		
Age, years	73.7 ± 5.8	73.5 ± 5.3	72.4 ± 5.2	72.8 ± 5.3	0.029	–
Women, n(%)	164 (53.4)	115 (62.8)	93 (45.4)	184 (63.0)	< 0.001	3,4
BMI, kg/m^2^	23.0 ± 3.3	23.4 ± 2.9	23.2 ± 3.2	23.1 ± 3.1	0.624	–
**Medication status**						
Hypertension, n(%)	149 (48.9)	89 (48.6)	80 (39.2)	120 (41.2)	0.067	
Psychotropic, n(%)	10 (3.3)	3 (1.6)	3 (1.5)	5 (1.7)	0.427	
Diabetes, n(%)	42 (13.8)	21 (11.5)	23 (11.3)	26 (8.9)	0.327	
Sleeping pills, n(%)	28 (9.2)	18 (9.8)	10 (4.9)	18 (6.2)	0.147	
**Medical history**						
Lower back pain, n(%)	70 (23.0)	42 (23.0)	41 (20.1)	50 (17.2)	0.290	
Knee pain, n(%)	47 (15.4)	24 (13.2)	25 (12.3)	38 (13.1)	0.739	
Hip pain, n(%)	11 (3.6)	7 (3.8)	5 (2.5)	9 (3.1)	0.861	
Alcohol consumption (drinker), n(%)	104 (34.1)	67 (36.6)	87 (42.6)	112 (38.5)	0.264	
Current smoker, n(%)	22 (7.2)	10 (5.5)	23 (11.3)	18 (6.2)	0.108	
GDS score, points	4.3 ± 2.9	3.2 ± 2.5	3.7 ± 2.8	2.7 ± 2.5	< 0.001	2,4 < 1, 4 < 3
PSQI score, points	5.6 ± 3.1	5.1 ± 3.0	4.9 ± 2.9	4.9 ± 3.2	0.024	4 < 1
**LSNS score, points**	12.9 ± 3.6	21.1 ± 2.8	13.6 ± 3.2	21.9 ± 3.3	< 0.001	1,3 < 2,4
Family ties, points	7.3 ± 2.2	10.9 ± 1.9	7.4 ± 2.1	11.2 ± 2.0	< 0.001	1,3 < 2,4
Friendship ties, points	5.7 ± 2.8	10.2 ± 1.9	6.2 ± 2.5	10.7 ± 2.2	< 0.001	1,3 < 2,4
**PASE score, points**	76.7 ± 28.0	81.1 ± 26.6	158.7 ± 40.3	175.3 ± 56.6	< 0.001	1,2 < 3,4, 3 < 4
Leisure-time physical activity, points	13.5 ± 13.8	14.9 ± 13.7	28.3 ± 24.7	32.9 ± 30.8	< 0.001	1,2 < 3,4
Housework physical activity, points	60.6 ± 27.9	64.7 ± 27.8	102.8 ± 30.8	109.2 ± 32.6	< 0.001	1,2 < 3,4
Work-related physical activity, points	2.6 ± 10.1	1.6 ± 5.1	27.6 ± 50.4	33.2 ± 56.4	< 0.001	1,2 < 3,4
**Physical functions**^**a**^						
Grip strength, kg	27.37 ± 0.28	27.88 ± 0.37	27.82 ± 0.34	28.58 ± 0.29	0.029	1 < 4
One-leg standing duration, sec	31.20 ± 1.26	34.64 ± 1.63	36.93 ± 1.51	36.03 ± 1.28	0.013	1 < 3,4
Timed up and go test, sec	6.20 ± 0.07	6.02 ± 0.09	5.98 ± 0.09	5.86 ± 0.07	0.011	4 < 1
5-m walk test, sec	3.73 ± 0.04	3.61 ± 0.05	3.70 ± 0.05	3.58 ± 0.04	0.027	4 < 1
5 times sit-to-stand test, sec	3.73 ± 0.04	3.61 ± 0.05	3.70 ± 0.05	3.58 ± 0.04	0.005	2,4 < 1

Note: *SD* standard deviation, *BMI* Body mass index, *GDS* geriatric depression scale, *LSNS* Lubben social network scale, *PASE* physical activity scale for the elderly; ^a^ low activity group with low social relationships (*n* = 260); low activity group with high social relationships (*n* = 152); high activity group with low social relationships (*n* = 179); and high activity group with high social relationships (*n* = 251) due to missing data

In terms of physical functions, the high activity group with high social relationships shows significantly positive value compared to the low activity group with low social relationships on all of the physical functions (Table 1). Moreover, compared to the low activity group with low social relationships, the high activity group with low social relationships on the one-leg standing duration shows a longer duration, and low activity group with high social relationships on the 5 times sit-to-stand test shows shorter duration (all *P*s < 0.05).

Table 2 presents the association between physical activity and social relationships on sleep disorder. In the unadjusted model and Model 1, both variables show linear trends regarding the high risk of sleep disorder. However, the linear trends are significantly different in Model 2, which features adjustments for the other variable (i.e., physical activity or social relationships, depending on the variable being measured; Table 2). In Model 1, the unadjusted model, and Model 2, the high risk of sleep disorder is significantly lower in the third tertile of both physical activity and social relationships than in the first tertile of the respective variable.

**Table 2:** Association between physical activity, social relationship, and sleep disorder

Variables	No. of sleep disorder, n (%)		Odds ratio (95% CI)					
			Unadjusted		Model 1		Model 2	
**Physical activity**			Trend *P* value = 0.008		Trend *P* value = 0.049		Trend *P* value = 0.126	
1st tertile	133	(43.5)	1.000	(Ref)	1.000	(Ref)	1.000	(Ref)
2nd tertile	107	(35.1)	0.703	(0.507–0.974)	0.766	(0.542–1.083)	0.784	(0.553–1.109)
3rd tertile	97	(31.7)	0.604	(0.434–0.840)	0.645	(0.453–0.919)	0.694	(0.484–0.997)
**Social relationship**			Trend *P* value = 0.042		Trend *P* value = 0.047		Trend *P* value = 0.121	
1st tertile	137	(42.0)	1.000	(Ref)	1.000	(Ref)	1.000	(Ref)
2nd tertile	101	(34.9)	0.741	(0.535–1.028)	0.777	(0.549–1.101)	0.799	(0.563–1.134)
3rd tertile	99	(32.8)	0.673	(0.486–0.932)	0.644	(0.453–0.915)	0.690	(0.481–0.988)

Note: sleep disorder means PSQI global score > 5 pts.; 95% CI, 95% confidence interval; pts., points; Ref, reference; LSNS, Lubben social network scale; PASE, physical activity scale for the elderly; Model 1, adjusted for age, sex, BMI, take medication of hypertension, sleeping pills, had a smoking and drinking habit, and depressive syndrome; Model 2, Model 1 and additional adjustments for physical activity variables when examine social relationship. Also, additional adjustments for social relationship variables when examine physical activity

Table 3 shows the combined association between physical activity and social relationships on sleep disorder. The only significant difference in Model 1 concerns the high activity group with high social relationships, which features a 41.5% lower risk of sleep disorder than the low activity group with low social relationships. The other groups show no significant differences from the low activity group with low social relationships (Table 3).

**Table 3:** Association between combined physical activity and social relationship, and sleep disorder

Variables			No. of sleep disorder, n (%)		Odds ratio (95% CI)			
					Unadjusted		Model 1	
Physical activity		Social relationship						
low (0–115 pts)	&	low (2–17 pts)	126	(44.1)	1.000	(Ref)	1.000	(Ref)
low (0–115 pts)	&	high (18–30 pts)	61	(35.7)	0.704	(0.477–1.040)	0.672	(0.442–1.022)
high (116–425 pts)	&	low (1–17 pts)	64	(33.7)	0.645	(0.441–0.944)	0.697	(0.465–1.043)
high (116–462 pts)	&	high (18–30 pts)	86	(31.9)	0.594	(0.420–0.839)	0.585	(0.404–0.847)

Note: sleep disorder means PSQI global score > 5 points; 95% CI, 95% confidence interval; pts., points; Ref, reference; Model 1, adjusted for age, sex, BMI, take medication of hypertension, sleeping pills, had a smoking and drinking habit, and depressive syndrome

**Fig. 1:**
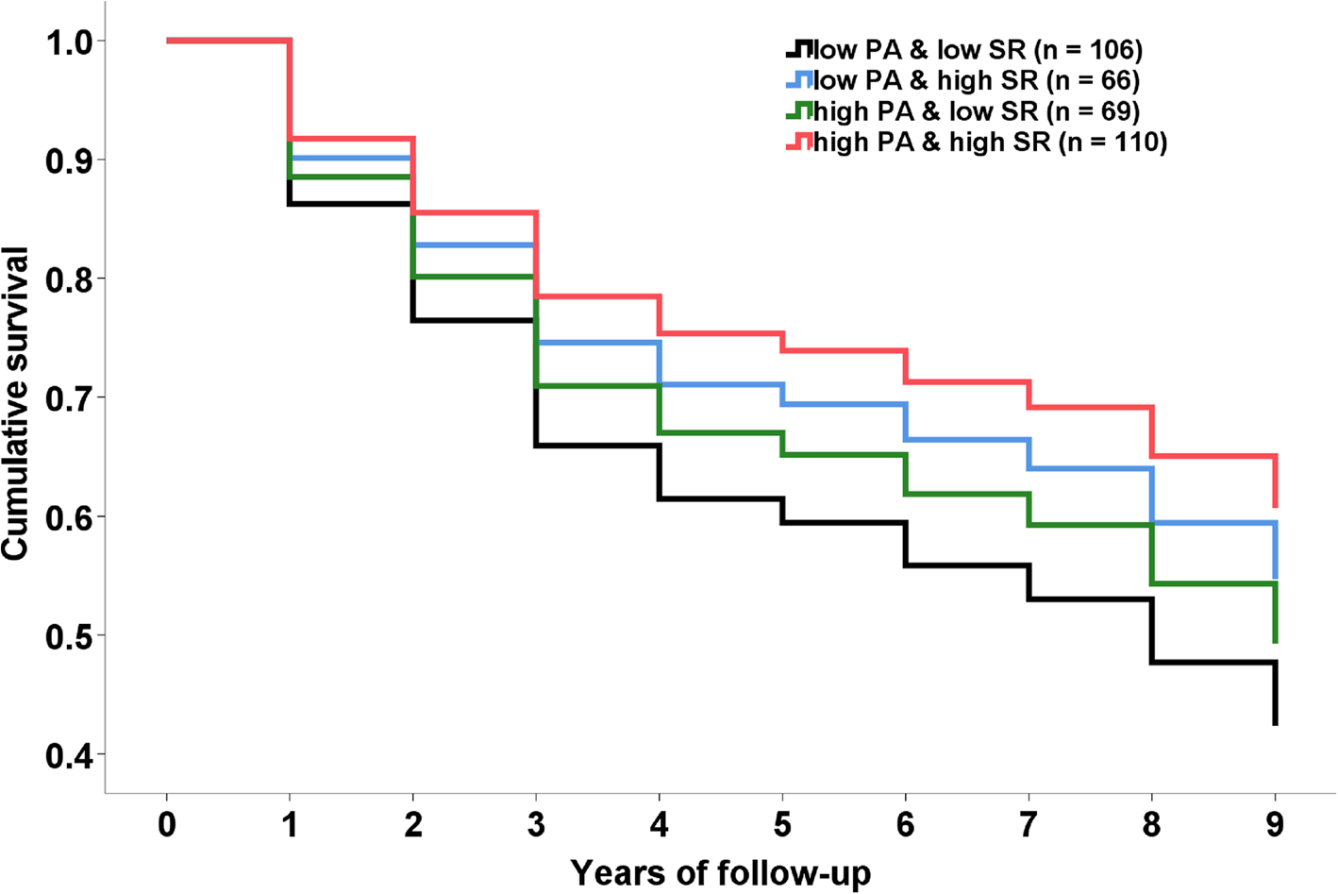
The adjusted Kaplan-Meier curves for sleep disorder by combination of physical activity and social relationships among all subjects in Model 1. Survival curves were adjusted for age, gender, body mass index, use of hypertension and sleeping medication, smoking and drinking habits, and depressive syndrome. PA, physical activity; SR, social relationship

The mean age of the sample in Study 2 was 72.4 ± 5.0 years, BMI was 23.1 ± 2.9 kg/m^2^, and 49.6% of the participants were female. During a mean follow-up of 3.7 years (1283 person-years), sleep disorder occurrence was 96 participants (27.4%). Figure 1 and Table 4 show the combined effect of physical activity and social relationships on sleep disorder. Figure 1 shows the adjusted Kaplan-Meier curves for sleep disorder by group among all participants in Model 1. Table 4 shows that the HRs of the high activity group with high social relationships are significantly lower than that of the low activity group with low social relationships in Model 1.

**Table 4:** Hazard ratios for sleep disorder by each group

Variables			No. of sample	No. of person-years	No. of sleep disorder	Hazard ratio (95% CI)			
						Unadjusted		Model 1	
Physical activity		Social relationship							
low (0–115 pts)	&	low (2–17 pts)	106	417	38 (35.8%)	1.000	(Ref)	1.000	(Ref)
low (0–115 pts)	&	high (18–30 pts)	66	280	18 (26.1%)	0.648	(0.369–1.140)	0.675	(0.379–1.202)
high (116–425 pts)	&	low (1–17 pts)	69	258	19 (28.8%)	0.772	(0.445–1.340)	0.803	(0.458–1.407)
high (116–462 pts)	&	high (18–30 pts)	110	328	21 (19.1%)	0.595	(0.349–1.015)	0.564	(0.327–0.974)

Note: sleep disorder means PSQI global score > 5 points; 95% CI, 95% confidence interval; pts., points; Ref, reference; Model 1, adjusted for age, sex, BMI, take medication of hypertension, sleeping pills, had a smoking and drinking habit, and depressive syndrome

## Discussion

This study examined sleep disorder among older adults in terms of its association with daily physical activity and social relationships, both individually and in combination with each other using cross-sectional and longitudinal studies. Our study showed that a high level of physical activity and high level of social relationships may, independently of each other, positively affect sleep quality (Table 2). This result is similar to previous findings that low levels of physical activity and narrow ranges of social relationships are related to poor sleep quality in older adults [9, 20]. However, unlike these previous studies, we adjusted for the effect of one variable when measuring the other. The risk of sleep disorder was significantly lower in the high activity group with high social relationships, compared with the low activity group with low social relationships (Tables 3 and 4). These results do not support our hypothesis that high levels of one variable with low levels of the other (e.g., low activity people with high social relationships) results in a lower prevalence of sleep disorder than low activity levels and a narrow range of social relationships.

Sleep disorder and depressive syndrome are strongly linked [36], and the rates of both are significantly higher among older adults than younger people [37]. Overall, 83% of patients with depression have experienced some form of sleep disturbance [36] and, although the effects of daily sleep quality on next-day mood are larger than the effects of daily mood on sleep quality, sleep quality and mood share a bilateral relationship [38]. Some previous studies have shown that strategies for regulating mood control can improve sleep quality [39]. In this study, although no significant differences were observed, the groups with low levels on one variable (i.e., the low activity group with high social relationships and the high activity group with low social relationships) showed 14–26% lower depressive syndrome scores (i.e., GDS score; Table 1) than the low activity group with low social relationships. Meanwhile, the high activity group with high social relationships showed a significantly lower value than the low activity group with low social relationships (37% lower) and the high activity group with low social relationships (27% lower) in Study 1. The significant difference in sleep disorder between the low activity group with low social relationships and the high activity group with low social relationships disappeared after full adjustment for covariates, including depressive syndrome. However, the difference between the low activity group with low social relationships and the high activity group with high social relationships remained significant (Table 3). Similarly, Model 1 in Study 2 shows a significant difference in sleep disorders between the low activity group with low social relationships and the high activity group with high social relationships (Table 4).

Researchers have highlighted several potential mechanisms by which physical activity affects sleep quality, including energy conservation, body and central nervous system temperature increases, and anxiety reduction [40–42]. In addition, some previous studies have mentioned that the relationship between physical activity and sleep disturbances might be mediated by physical functioning [15, 16]. High levels of both variables indicated significantly better health across all physical functions compared with low levels. These results demonstrate that high levels of physical activity and sleep disturbance may have a positive effect on sleep quality mediating physical functions (Tables 3, 4; Fig. 1).

Meanwhile, the effects of social relationships on sleep quality are not fully understood. Theoretically, however, strong social relationships provide an evolutionarily adaptive function, creating a safe environment in which sleeping persons feel they are protected by others from dangers (e.g., in an evolutionary context, predators and enemies) [20]. Recent studies have also hypothesized that social isolation causes chronic stress [17], directly affects anxiety, and decreases sleep satisfaction [20, 21]. Social isolation is related to lower physiological functioning and higher risks of physical disorders [17]. In particular, addressing deficits in terms of social relationships and physiological activity may directly arrest early progression toward chronic diseases, and could also delay disease onset and lessen disease burden in later life [17]. The above findings suggest that physical activity and social relationships produce overlapping, synergistic anti-anxiety and anti-depressant effects.

In a previous study, the present authors revealed that communal exercise has a lower hazard ratio for five-year morality than no exercise [43]. The present study suggests that physical activity and exercising with others [44, 45] may have a synergistic effect on not only physical functions, but also mental health and sleep quality in older adults.

A previous epidemiological study showed that intellectual, social, and recreational activities are correlated with cognitive function [23]. Indeed, an experimental study revealed that low-intensity exercise with social interaction not only has positive effects on sleep quality, but also improves cognitive function, particularly attention ability, in older adults [22]. It has been postulated that exposure to social relationships and engaging in physical activity increase blood flow to the brain, improving neuronal function and facilitating deep sleep [22, 23]. We suggest that future studies should examine whether the effects of physical activity and social interaction on older adults’ sleep quality are mediated by improved cognitive function; methods such as polysomnography and functional near-infrared spectroscopy could be used to perform this examination.

Our study has several limitations. First, although this study examined the lower prevalence of sleep disorder among individuals who are active and have a wide range of social relationships (i.e., lacking in neither variable), we could explore the causal relationship; however, we could not explain the mechanism. Thus, this aspect warrants further investigation through an experimental study or an intervention study, for example. Second, we did not examine whether supportive and aversive social relationships have different effects on sleep disorder. In this study, we only focused on supportive relationships because a previous study revealed no significant association between aversive relationships and sleep quality [20]. Generally, the LSNS cut-off is used for social isolation with less than 12 points [27], and high scores on the LSNS indicate abundant social networks [46]. However, since we aimed to ensure statistical power and for ease in interpretation of the results, we used continuous data divided into median or tertile [28, 29]. Especially, the PASE has subscales such as exercise types (walking, low-, moderate-, vigorous-intensity exercises), household activity, and work-related activity [12, 26]. Nevertheless, further studies should consider the kind of activity and adverse relationships in this regard. Third, all variables were assessed using self-report scales; thus, our findings may have been influenced by recall and reporting bias. Fourth, physical activity and social relationship factors may be influenced by individual differences. Thus, future studies should consider gender differences and implement stratification in terms of physical function and the presence of depressive syndrome. Further, the type of physical activity and/or social relationship is not only affected by individual differences, but also by cultural and/or regional differences [27]. Thus, the results of the present study need to be carefully interpreted. Finally, although a previous epidemiological study involving a Japanese population [1] reported the same prevalence of sleep disorder (36.2%) as in the present study, the generalizability of our results is uncertain because of the small sample size.

## Conclusions

In conclusion, high levels of physical activity and social relationships are independently related to good sleep quality. Our findings suggest that being highly active with a wide range of social relationships is favorably associated with sleep quality. However, a high level of one variable with a low level of the other, has not been confirmed to improve sleep quality among older adults. These results suggest the importance of considering both, physical activity and social relationships, when seeking to improve sleep quality among older adults.

## Data Availability

The datasets used and/or analyzed during the current study are available from the corresponding author on reasonable request.
